# Changes in Affect Integration and Internalizing Symptoms After Time-Limited Intersubjective Child Psychotherapy—A Pilot Study

**DOI:** 10.3389/fpsyg.2022.906416

**Published:** 2022-07-08

**Authors:** Charlotte Fiskum, Tonje G. Andersen, Unni T. Johns, Karl Jacobsen

**Affiliations:** ^1^Department of Psychology, Norwegian University of Science and Technology, Trondheim, Norway; ^2^Child and Adolescent Mental Health Services, Akershus University Hospital, Oslo, Norway; ^3^Department of Psychology, University of Oslo, Oslo, Norway

**Keywords:** psychodynamic therapy, parental work, play therapy, affect integration, regulation, mentalization, right-brain psychotherapy

## Abstract

Time-limited intersubjective child-psychotherapy (TIC) is an intensive, structured right-brain oriented therapeutic approach for children and caregivers aimed at both symptom reduction and strengthening core developmental processes such as affect integration. This is the first study to investigate changes in internalizing symptoms and affect integration after TIC. Thirty-three children between 9 and 13 years with internalizing difficulties were assessed after 10 h of TIC. Internalizing symptoms were assessed through the child behavior checklist and affect integration with the affect consciousness interview (ACI). Scores from the children with internalizing difficulties were modeled in a mixed linear model along with data from a control group without internalizing difficulties (*n* = 24) to control for effects of time and learning. Affect integration increased significantly from time 1 to time 2 in the children with internalizing problems, but not in the control children. Internalizing symptoms were also significantly reduced in the children with internalizing difficulties from time 1 to time 2. The results indicate that TIC may contribute to a decrease in symptoms along with an increase in affect integration in children with internalizing difficulties, making the children better able to notice, tolerate and communicate affective states. This can, in turn, aid development and day-to-day regulation and interactions. The article explores key elements of TIC, such as affective attunement, relational repair, and work with caregivers using one of the individual therapies in the study as an example. The article argues that TIC represents a right-hemisphere to right-hemisphere therapeutic approach to healing that can both enhance important common therapeutic factors such a relation and alliance and bring about growth-promoting change.

## Introduction

Time-limited intersubjective child psychotherapy (TIC) is a therapeutic method rooted in psychodynamic theory and phenomenology, integrating knowledge and concepts from empirical infant psychology and transactional psychology (Sameroff, 2009; Hansen, 2012; Johns and Svendsen, 2016). Originally inspired by Stern’s, Trevarthen’s, and Tronick’s work (Trevarthen, 1989; Tronick et al., 1998; Stern, 2000; Trevarthen and Aitken, 2001), TIC has been developed in Norway over the last decades, serving as an inspiration for time-limited mentalization based therapy for children (MBT-C) (Midgley et al., 2017). As in MBT-C, the focus in TIC is on elements that facilitate healthy developmental processes and growth rather than on symptom reduction alone. What differentiates TIC from MBT-C is a wider focus on processes presumed to underlie and support the capacity to mentalize such as regulation of affect and arousal and capacity for intersubjective transactions between the child and caregivers. Further, while MBT-C is more focused toward left-lateralized functions, TIC is more focused on right-lateralized functions, in line with Schore’s right-brain psychotherapeutic perspective (Schore, 2019).

In TIC, present difficulties are seen as arising from disturbances or shortcomings in the child’s developmental processes, the developmental support system, or the transactions between the two. Therefore, TIC aims not merely to reduce present-day symptoms but, just as importantly, to facilitate, enhance, strengthen, or (re)start core developmental processes within the child and its caregivers. Particular weight is given to the ability to part-take in meaningful intersubjective transactions (i.e., the dynamic sharing of subjective experiences between minds) and core functions or processes related to this, such as agency, self-other-regulation, and affect integration. The importance of intersubjective transactions stems from empirical studies of co-regulation in infants and children (Tronick et al., 1998; Stern, 2000; Trevarthen and Aitken, 2001; Johns and Svendsen, 2016) as well as insights from Sameroff’s transactional model (Sameroff, 2009). Children and caregivers constitute a dynamical, transactional system that is continuously created and affected by the contributions of each participant. In such an expanded state of intersubjective “being-together” one can view partners in a dyad as creating a larger consciousness and regulating space than when alone, allowing the less mature or organized nervous system to reorganize in synchronicity with the more mature or more organized nervous system (Tronick et al., 1998).

### Key Elements of Time-Limited Intersubjective Child Psychotherapy

First, the child’s developmental history and intersubjective capacity are assessed to establish fit with TIC and increase the precision and efficacy of the interventions (Johns and Svendsen, 2016). The assessment usually involves three sessions with the child, allowing the therapist to pinpoint important processes that need particular attention. Here the ability to sustain and share attention, take turns, play, regulate arousal and affect, and participate in a relational exchange is considered essential. Initial assessment of affect integration (Monsen et al., 1996; Taarvig et al., 2015) can help pinpoint particularly difficult affects for the child, allowing them to be addressed in therapy. Caregivers’ ability to sensitively attune to and support their child is also assessed in three parallel sessions. After the assessment, an explicit and agreed-upon focus for therapy is formulated with the child and caregivers. The focus is stated as a metaphor that points to developmental possibilities and aims to evoke interest, recognition, motivation, and participation in therapy. Examples include “going hunting for your buried treasures” and “helping you sing all the notes in your song.” The metaphor should mirror the child’s experiential world as presented by the child as well as the caregivers’ understanding of the child, while aspects of togetherness emphasize that the child, caregivers, and therapist all work together (Johns and Svendsen, 2016).

The structure of TIC is designed to strengthen the child’s agency, understanding, and meaningful ownership of their therapeutic process, as well as the alliance (Johns and Svendsen, 2016). The emphasis on time-limitation aims to make the work both directed and understandable, with a clear beginning, middle, and end, usually spread over 12 weekly therapy sessions. A child can receive more than one time-limited intervention, if necessary, but each must follow the same structure and contain an agreed-upon focus and endpoint. A therapy calendar with a blank space for the child to draw or write something after each session functions as visual support, time-keeper, and narrative record for the child, therapist, and caregivers of what has happened so far, and where one is relative to the end of therapy (Johns and Svendsen, 2016).

The therapeutic principles of TIC are based on psychodynamic theory (Winnicott, 2005) and empirical studies of co-regulation and development in infants and children (Tronick et al., 1998; Stern, 2000; Trevarthen and Aitken, 2001; Johns and Svendsen, 2016). Young children are dependent on caregivers for survival and regulation. In addition, the caregiver assists the child in a gradual awakening of awareness and recognition of internal states, making sensations such as hunger, tiredness, and prototypical affects like pleasure or displeasure become realized and organized in the child’s mind and body. Through experiences of affect attunement, the child implicitly learns that what is inside belongs to a whole (me) that can be grasped, known, shared, tolerated, and regulated. These experiences include the integration of bodily and mental experiences associated with increasingly complex categories of affect. Studies of infants show the importance of multimodal and synchronized stimuli that provide an embodied and enveloping experience regarding intensity, timing, and affective tone (Trevarthen, 1989; Stern, 2000). Therefore, the dialogue in TIC relies on sensitive, multimodal mirroring of affect and arousal to strengthen the integration of experiences.

Different therapeutic activities tailored to each child’s developmental needs can be introduced in TIC to help the child explore and integrate new ways of being and regulating (Johns and Svendsen, 2016). For a child struggling with sustained or shared attention, this may include jointly attending to activities the child shows interest in, such as play, games, videos, music, or drawings, thereby expanding moments of shared attention and mental connection leading to increased subjective awareness in the child (Jacobsen and Svendsen, 2010). The ability to take turns can be expanded by throwing a ball back and forth, playing board games, or musical or rhythmic play and improvisation. In some cases, children with a low tolerance for arousal may need help to expand their window of tolerance (Siegel, 1999; Ogden and Minton, 2000) or gain experience regulating arousal up and down (Jacobsen and Svendsen, 2010). Hyper-aroused children may need co-regulation from a responsive and regulated therapist using their voice, body, music, and activities or soothing rhythms to downregulate excessive physiological arousal (Dana, 2018). In the same way, hypo-aroused children can be awakened and stimulated by introducing energetic music or vigorous physical play within a safe relational frame (Eide-Midtsand, 2017). All the while, the therapist recognizes, mirrors, names, expands, or modulates affects encountered in therapy to enhance the integration of affective experience.

Alongside the individual, child-centered work, caregivers receive parallel sessions focused on strengthening their reflective function (Haugvik, 2012) and their ability to provide appropriate developmental support for their child. Because of the importance of healthy transactional exchanges between children and caregivers, the capacity to attune to, connect with, understand, and regulate the child is particularly pertinent.

Crucially, TIC is aimed not only at symptom-reduction but is also explicitly concerned with strengthening or (re)starting processes that can help place children and caregivers on more developmentally healthy transactional paths. One such process considered highly important for healthy development and meaningful transactions with others is affect integration (Monsen et al., 1996; Jacobsen and Svendsen, 2010; Solbakken et al., 2011).

### The Importance of Affect and Affect Integration

Affects, such as emotions and moods, can be understood as “processes of establishing, maintaining and disrupting the relationships between the person and the internal and external environment when such relations are significant to the individual” (Campos et al., 1989). In this perspective affects imbue mental and internal or external physical events with significance by vitalizing, magnifying, and clarifying events and relationships (Saarni et al., 1998; Campos et al., 2004). Affects are also associated with behavioral tendencies or impulses of approach or avoidance, and they represent a powerful means of communication of needs and desires (Campos et al., 1989, 2011; Monsen, 1990; Jacobsen and Svendsen, 2010; Svendsen and Jacobsen, 2013). For instance, anger can mobilize energy to overcome obstacles or to set or communicate clear boundaries with others. Likewise, feelings or expressions of joy invigorate or encourage the person (and those around) to keep doing what they are doing, while sadness communicates a loss and accompanying need for support and comfort (Campos et al., 1989).

*Affect integration* means that the vitality or motivational force of affect is integrated with cognitive and behavioral processes, thereby becoming more apparent and more understandable to the individual and those around (Monsen et al., 1996; Jacobsen and Svendsen, 2010; Solbakken et al., 2011; Taarvig et al., 2015). While emotion regulation often relates to the processes in which thoughts or behaviors regulate emotions, affect integration also concerns how affect influences and regulates other domains of function, including cognitive, motivational, behavioral, and relational processes (Monsen, 1990; Taarvig et al., 2015). Poorly integrated affect may present not as a source of meaningful information but rather as undifferentiated agitation or arousal lacking clarity of cause or resolution. Poorly integrated affect may also result in somatic symptoms or less adaptive regulation of arousal and behavior (Lane and Schwartz, 1987; Jacobsen and Svendsen, 2010). Furthermore, difficulties with affect integration can result in impoverished, ambiguous, or misleading interpersonal communication and interpersonal problems (Jacobsen and Svendsen, 2010; Campos et al., 2011; Svendsen and Jacobsen, 2013).

The development of affect integration depends on adequate attunement and satisfaction of affective needs within significant early relationships (Monsen, 1990; Jacobsen and Svendsen, 2010; Lane et al., 2015; Murphy et al., 2017). If, however, affects and needs remain consistently unseen, unmet, or misinterpreted due to dispositional, relational, or contextual factors, integration can suffer and can be supported or restarted through therapy (Monsen and Monsen, 1999; Jacobsen and Svendsen, 2010).

### Previous Studies and Aims of the Current Study

Difficulties with affect integration have been related to internalizing problems in children (Taarvig et al., 2015, 2016). In support of this, an earlier analysis of the children in the current study (Fiskum et al., 2021) showed significant differences in affect integration between children with internalizing difficulties and non-internalizing controls before therapy. Specifically, the children with internalizing difficulties were less aware of their affect, had a lower tolerance for affect, and found it more challenging to express affect to others. These differences were widespread and held for both positive and negative affects. Two previous studies have investigated the effects of TIC on aspects of affect integration. In the first, Haugvik and Johns (2008) found positive effects on symptoms and increased parent-reported clarity in the children’s emotional communication. Changes in affect integration following TIC have also been investigated in a small sample of the children in the present study in a video micro-analysis (Johns, 2018), indicating a positive effect on affect integration. The current study expands on this earlier work, looking at affect integration and internalizing symptoms after TIC in 33 children with internalizing difficulties. The children with internalizing difficulties are compared to a control group without internalizing difficulties (24 children) before and after therapy to control for effects of time and learning using a mixed linear model. Affect integration is assessed using the Affect Consciousness Interview (ACI) (Monsen et al., 1996; Taarvig et al., 2015) and internalizing symptoms with the Child Behavior Checklist (CBCL) (Achenbach and Rescorla, 2001). This article also explores aspects of TIC believed to help increase affect integration through an anonymized case.

## Materials and Methods

The study was a collaboration between two child and adolescent mental health outpatient clinics in two urban areas of Norway. The study’s background, procedures, and aims were explained to all participants, and parents provided written consent. The children were assessed with CBCL and interviewed with the ACI at the beginning of the project. The internalizing group was re-assessed after the completion of three assessment sessions and 10 weekly 45 min sessions of TIC. A group of children without internalizing difficulties was assessed at the same time points without any intervention to control for time and learning. The full period of data-collection spanned from spring 2014 to fall 2017, with the two groups assessed in parallel. For each individual, time 1 and time 2 measures were obtained 14–16 weeks apart. The study followed the Helsinki Declaration for research ethics (World Medical Association, 2013) and was approved by the Norwegian Regional Committees for Medical and Health Research Ethics, region north. The children were assessed in a quiet room by a senior clinical child psychologist with extensive experience working with children and affect integration. The assessment took place after the completion of a different part of the project involving heart rate measurements described previously (Fiskum et al., 2019).

### Subjects

#### Internalizing Group

The study recruited 9–13-year-old children through information to local social and psychiatric services, general practitioner doctors, school health services, and families referred to the participating clinics. Inclusion was based on a description of clinically significant internalizing difficulties (depressive, anxious, or mixed symptoms) and elevated scores of internalizing symptoms on the child behavior checklist (CBCL) (Achenbach and Rescorla, 2001) in the clinical or borderline clinical area. Exclusion criteria were suspected or confirmed learning difficulties, severe psychiatric disorders (e.g., psychosis), symptoms indicating predominantly externalizing rather than internalizing difficulties, and living in a family situation too unstable or conflictual to commit to weekly therapy. Forty children were considered for inclusion. The subjects’ referral and history as given by the parents were evaluated by two experienced clinical child psychologists and deemed to present with internalizing difficulties, and not fulfilling exclusion criteria. One child was too young, and four did not show elevated CBCL-scores. One child was not included due to scheduling problems, and one child was excluded due to possible symptoms of psychosis. The final group consisted of 33 children (18 girls).

#### Control Group

Inclusion in the control group called for children aged 9–13 with normal-range CBCL-scores and no history of referral to mental health services, including no suspicion of learning disabilities or any suspected or diagnosed mental health problems. The controls were recruited through information to parents at local schools and convenience sampling. Twenty-eight children were considered for inclusion. One child was excluded due to an earlier referral to mental health services on suspicion of developmental disorder. Two were excluded due to elevated CBCL-scores, and one child could not participate because of scheduling difficulties. Twenty-four children were included in the control group (13 girls).

#### Demographic Characteristics

Ten children in the internalizing group lived in Oslo. The remainder of the children in the study were from Trondheim. Both are urban areas of Norway. The mean age at time 1 was 10.91 years (SD 1.18) for the children with internalizing problems and 10.23 years (SD 1.19) for the controls. The children in the control group were, on average, a few months younger than the children in the internalizing group (Mann Whitney *U* = 259.0, *p* = 0.027). The sample had 53 children with Norwegian backgrounds. The rest had multicultural backgrounds (Norwegian-Asian and Norwegian-African), evenly distributed between the groups. In the internalizing group, 20 fathers and 24 mothers had completed at least 2 years in university or college, compared to 20 fathers and 22 mothers in the control group. This difference in parental education was non-significant for maternal (*U* = 320.0, *p* = 0.110) and paternal education (*U* = 275.0, *p* = 0.092).

### Therapeutic Intervention

Experienced child psychologists and graduate-level clinical psychology students gave the children in the internalizing group TIC according to the TIC-handbook (Johns and Svendsen, 2016). The students received training in TIC and weekly supervision by clinical specialists with extensive experience with TIC, based on direct observation of therapy through one-way- mirrors or videotapes. In all of the cases two therapists worked together as a team, with one therapist working individually with the child, the other with the caregivers. All therapies followed the same structure. This structure included three assessment sessions with the child and caregivers separately, performed by the child and caregiver-therapists respectively. The assessments focused on the clinical and developmental history of the child and their capacity for basic processes underlying intersubjectivity. After the assessment, the child, caregiver(s), and therapists agreed on a shared focus for therapy. The children then received 10 weekly therapy sessions, segmented into a clear beginning, middle, and end of therapy. Each segment of therapy was conducted with the child and caregivers separately, with 2–3 shared sessions and a concluding shared summary-session at the end. An anonymized example of TIC therapy illustrating salient aspects of TIC is included in the discussion, based on video recordings, therapy records, and ACI scores from one of the therapies in the project.

### Instruments and Procedures

#### The Child Behavior Checklist

The Child Behavior Checklist (CBCL) (Achenbach and Rescorla, 2001) is a questionnaire for parents assessing common symptoms of mental health problems in childhood standardized against the DSM-IV (Achenbach and Dumenci, 2001). The descriptive value of each symptom is rated on a 3-point Likert scale ranging from Not True to Very True/Often True. The current study focused on the internalizing subscale (32 items, Cronbach’s α = 0.90).

#### The Affect Consciousness Interview

Affect integration was assessed using a version of the ACI (Monsen et al., 1996; Taarvig et al., 2015) for children (Monsen et al., 2013) assessing the 10 following affective categories: (1) Interest/Excitement, (2) Enjoyment/Joy, (3) Fear/Panic, (4) Anger/Rage, (5) Disgust/Contempt, (6) Shame/Humiliation, (7) Sadness/Despair, (8) Envy/Jealousy, (9) Guilt/Remorse, and (10) Tenderness/Care. Initial experience with the ACI with children in our age group showed that Disgust/Contempt was challenging to respond to for many, and this category was therefore not included.

The interview focused on each affect by asking the child to describe a scene or situation in which they could remember feeling the affect in question. The interview then explored the focal affect across four dimensions; Awareness, Tolerance, Emotional Expressivity, and Conceptual Expressivity. *Awareness* describes to what degree and extent the person is aware of, attentive to, and recognizes bodily and psychological cues of affect. When assessing awareness, the interviewer asked the child how they would notice that they were experiencing a particular affect (like joy), drawing attention to both mental and physical aspects of affective experiences. *Tolerance* describes how well the child tolerates the affect in question, the availability of strategies (voluntary and involuntary) to regulate and manage the affect, and how the affect is used to extract meaningful information about the self, other people, and the outer world. For assessment of tolerance, the interviewer asked how it is for the child to feel the affect, how well they can tolerate, handle, and regulate the affect, and to what degree they can use the affect as a source of information. *Emotional Expressivity* describes the capacity to acknowledge and display clear, nuanced, emotional expressions through facial expressions, vocal pitch/tone, intonation, or bodily language. The final dimension, *Conceptual Expressivity*, describes how well the child can articulate different affects in an unambiguous and differentiated fashion in words (Taarvig et al., 2015). For these two dimensions, the interviewer asks the child to describe how they would express the affect in focus when alone or with significant and unknown others, first through non-verbal communication and then verbally.

Each affect was scored on a 9-point scale across the four separate dimensions (resulting in 4 scores per affect). A score of 1 is the lowest possible score indicating no/very little integration, while the highest possible score (9) indicates highly mature integration. Integration of each individual affect was calculated as the mean score across the four dimensions. Next, a score for each of the four dimensions was calculated across all affects, representing overall affective awareness, tolerance, and emotional and conceptual expressivity. Finally, an overarching Affect Consciousness score was calculated from all scores, reflecting overall levels of affect integration (Taarvig et al., 2015).

The ACI has displayed good psychometric properties and external validity (Solbakken et al., 2011; Taarvig et al., 2015). A previous study of 11-year-old children with anxiety (mean age 11.5) saw scores below 4 as an indication of problems (Taarvig et al., 2016). As 10 of the children were assessed in a location where clinical status was implied and blinding of these cases was not possible, a second, blinded rater was used to minimize the risk of bias. The blind rater rescored nine of the interviews based on audiotapes. Interrater reliability between the two raters was assessed by a two-way random intraclass correlation coefficient which ranged from good (0.82) to excellent (0.99) with a mean correlation coefficient of 0.93, showing a very high consistency between the blinded rater and the original rater (Fiskum et al., 2021).

### Attrition From Time 1 to Time 2

Three children with internalizing difficulties did not show up for the assessment at time 2; for one child, the parents still filled out the CBCL. Two control children did not show up for the assessment at time 2; the parents still filled out the CBCL for both children. Scheduling difficulties were given as the reason for all children except one with internalizing difficulties who chose not to be reinterviewed.

### Statistical Analysis

The data was checked for extreme outliers, defined as a score more than three standard deviations away from the mean (z score ± 3.29). One data point was considered an outlier and removed. Change in affect integration and internalizing problems was evaluated using linear mixed random intercept models run in STATA 16.1. We specified subjects as a random factor and used restricted maximum likelihood estimation. Inspection of residuals indicated normality of distribution for errors. Group (internalizing/control), time (pre/post), and sex were entered as categorical independent variables, age as a continuous variable. A group × time interaction was entered to assess level and change in the dependent variables among the children with internalizing difficulties and controls. Interactions were interpreted using tests of estimated marginal effects. Alpha level was set to 0.05 *a priori*.

## Results

### Means and Estimated Marginal Means

Means and standard deviations of internalizing symptoms and affect integration at time 1 and time 2 is given in Table 1. Estimated marginal means and 95% confidence intervals from the linear mixed random intercept models are presented in Table 2.

**TABLE 1:** Means and standard deviations of internalizing symptoms and affect integration at time 1 and 2.

	Children with internalizing difficulties						Control children					
	Time 1			Time 2			Time 1			Time 2		
Measurement	*n*	*M*	SD	*n*	*M*	SD	*n*	*M*	SD	*n*	*M*	SD
Internalizing symptoms	33	68.7	9.0	32	60.78	9.68	24	46.92	8.53	24	47.29	8.72
Affect consciousness^a^	33	3.28	0.62	30	4.03	0.68	24	4.25	0.56	22	4.21	0.61
Awareness^b^	33	3.67	0.82	30	4.46	0.72	24	4.56	0.6	22	4.38	0.81
Tolerance	32	3.71	0.58	30	4.31	0.70	24	4.54	0.51	22	4.36	0.54
Emotional expressivity	33	3.51	0.67	30	4.36	0.77	24	4.69	0.64	22	4.72	0.62
Conceptual expressivity	33	2.3	0.67	30	2.98	0.91	24	3.2	0.79	22	3.37	0.8

*^a^Affect consciousness; mean of all scores on the affect consciousness interview.*

*^b^The dimensions Awareness, Tolerance, and Emotional and Conceptual Expressivity were scored as the mean for each dimension across all affects combined, Means and standard deviations from Time 1 has previously been presented (Fiskum et al., 2021).*

**TABLE 2:** Estimated marginal means and 95% confidence intervals of internalizing symptoms and affect integration at time 1 and 2.

	Children with internalizing difficulties				Control children			
	Time 1		Time 2		Time 1		Time 2	
Measurement	emMean	95% CI	emMean	95% CI	emMean	95% CI	emMean	95% CI
Internalizing symptoms	68.88	65.76–72.01	61.05**	57.89 – 64.21	46.7	43.02–50.38	47.07	43.4–50.75
Affect consciousness^a^	3.25	3.04–3.46	4.0**	3.79 – 4.22	4.29	4.04–4.54	4.22	3.96-4.47
Awareness^b^	3.65	3.39–3.9	4.43**	4.17 – 4.7	4.6	4.3–4.9	4.38	4.06–4.69
Tolerance	3.7	3.49–3.9	4.29**	4.08 – 4.5	4.56	4.32–4.8	4.36	4.12–4.61
Emotional expressivity	3.49	3.25–3.72	4.34**	4.09 – 4.58	4.72	4.45–5.0	4.73	4.44–5.02
Conceptual expressivity	2.26	2.0–2.52	2.96**	2.69-3.23	3.26	2.95–3.56	3.4	3.09–3.72

*Table displaying estimated marginal means (emMean) for the two groups at time 1 and time 2 along with 95% confidence intervals.*

*^a^Affect consciousness; mean of all scores on the affect consciousness interview.*

*^b^The dimensions Awareness, Tolerance, and Emotional and Conceptual Expressivity were scored as the mean for each dimension across all affects combined.*

** *Indicates that estimated marginal means were significantly different from time 1 to 2 at p > 0.001. Bold font Indicates that estimated marginal means were significantly different from time 1 to 2.*

### Linear Mixed Models: Interactions and Changes From Time 1 to Time 2

#### Internalizing Problems

The children with internalizing difficulties showed a significant change in Internalizing Problems as indicated by a significant group × time interaction (B = 8.21, 95% CI [3.31, 13.11], *p* = 0.001). Tests of marginal effects/means indicated a significant decrease in symptom severity from pre- to post-assessment in the patient group [χ^2^_(1)_ = 23.05, *p* < 0.001], but no significant change in the control group [χ^2^_(1)_ = 0.04, *p* = 0.843]. Marginal means from the patient and control groups were still significantly different at post-assessment [χ^2^_(1)_ = 31.03, *p* < 0.001], with higher symptoms in the internalizing group.

#### Affect Consciousness

The children with internalizing difficulties showed a significant change in Affect Consciousness as indicated by a significant group × time interaction (B = –0.82, 95% CI [–1.15, –0.49], *p* < 0.001). Tests of marginal effects/means indicated a significant increase in affect consciousness from pre- to post-assessment in the patient group [χ^2^_(1)_ = 46.3, *p* < 0.001], but no significant change in the control group [χ^2^_(1)_ = 0.29, *p* = 0.589]. Marginal means from the patient and control groups were not significantly different at post-assessment indicating similar levels of overall affect integration at time 2 [χ^2^_(1)_ = 1.52, *p* = 0.218].

#### Awareness

The children with internalizing difficulties showed a significant change in Awareness as indicated by a significant group × time interaction (B = –1.01, 95% CI [–1.42, –0.61], *p* < 0.001). Tests of marginal effects/means indicated a significant increase from pre- to post-assessment in the patient group [χ^2^_(1)_ = 34.39, *p* < 0.001], but no significant change in the control group [χ^2^_(1)_ = 2.05, *p* = 0.152]. Marginal means from the patient and control groups were not significantly different at post-assessment [χ^2^_(1)_ = 0.07, *p* = 0.786].

#### Tolerance

The children with internalizing difficulties showed a significant change in Tolerance as indicated by a significant group × time interaction (B = –0.79, 95% CI [–1.1, –0.49], *p* < 0.001). Tests of marginal effects/means indicated a significant increase from pre- to post-assessment in the patient group [χ^2^_(1)_ = 33.58, *p* < 0.001], but no significant change in the control group [χ^2^_(1)_ = 2.81, *p* = 0.094]. Marginal means from the patient and control groups were not significantly different at post-assessment [χ^2^_(1)_ = 0.18, *p* = 0.673].

#### Emotional Expressivity

The children with internalizing difficulties showed a significant change in Emotional Expressivity as indicated by a significant group × time interaction (B = –0.84, 95% CI [–1.21, –0.47], *p* < 0.001). Tests of marginal effects/means indicated a significant increase from pre- to post-assessment in the patient group [χ^2^_(1)_ = 47.88, *p* < 0.001], but no significant change in the control group [χ^2^_(1)_ = 0.00, *p* = 0.961]. Marginal means from the patient and control groups were still significantly different at post-assessment [χ^2^_(1)_ = 4.13, *p* = 0.042].

#### Conceptual Expressivity

The children with internalizing difficulties showed a significant change in Conceptual Expressivity as indicated by a significant group × time interaction (B = –0.55, 95% CI [–1.03, –0.07], *p* = 0.024). Tests of marginal effects/means indicated a significant increase from pre- to post-assessment in the patient group [χ^2^_(1)_ = 19.26, *p* < 0.001], but no significant change in the control group [χ^2^_(1)_ = 0.61, *p* = 0.436]. Marginal means from the patient and control groups were still significantly different at post-assessment [χ^2^_(1)_ = 4.32, *p* = 0.038].

**FIGURE 1:**
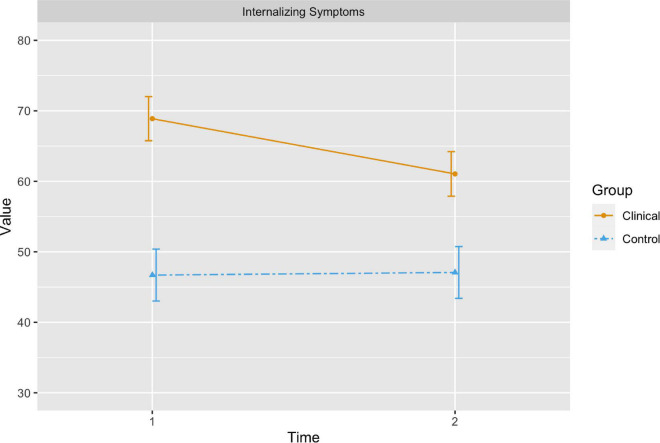
The orange (solid) line represents the change in estimated marginal means for internalizing symptoms in the children with internalizing difficulties from time 1 to time 2, while the blue (dashed) line represents changes in internalizing symptoms in the control group. The bars represent 95 percent confidence intervals.

**FIGURE 2:**
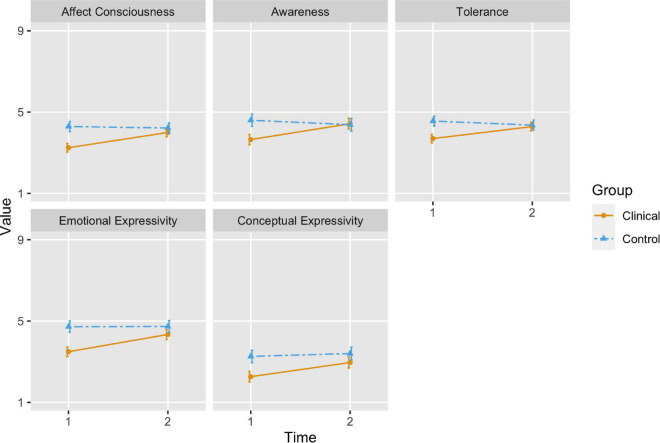
The orange (solid) line represents the change in estimated marginal means for affect integration scores in the children with internalizing difficulties from time 1 to time 2, while the blue (dashed) line represents changes in affect integration in the control group. The bars represent 95 percent confidence intervals.

Estimated marginal means and 95% confidence intervals are presented visually in Figures 1, 2.

## Discussion

### Changes in Internalizing Symptoms

The results showed that internalizing symptoms decreased significantly after TIC for the children with internalizing difficulties. There was no significant change in the symptom scores of the control group. As the study used children without internalizing difficulties as controls, the possibility of a natural remission of symptoms cannot be dismissed. Despite decreased symptoms, the children with internalizing difficulties still showed more internalizing symptoms than the control group after therapy. However, the focus in TIC is not merely on symptom reduction but also on strengthening and stimulating core developmental processes, such as affect integration, which may lead to greater symptom-reduction over time as development progresses (Johns and Svendsen, 2016). This focus is consistent with a growth-focus in psychotherapy (Schore, 2019).

### Changes in Affect Integration After Time-Limited Intersubjective Child Psychotherapy

The children with internalizing difficulties showed a significant increase in overall affect integration following TIC, as assessed by the affect consciousness score. They scored significantly higher across all four dimensions (awareness, tolerance, and emotional and conceptual expressivity) after TIC. There were no significant changes in affect integration for the control children. While the clinical children scored significantly lower than the healthy controls on all aspects of affect integration before treatment (Fiskum et al., 2021), the groups did not differ significantly in overall affect integration, awareness of, or tolerance for, affect after therapy. Furthermore, the ACI scores for the internalizing group moved out of the range considered low (scores below 4) after therapy for all dimensions except Conceptual Expressivity. Overall, the results indicate a significant increase in affect integration in children with internalizing difficulties after TIC. These changes in affect integration can positively influence development in several ways by making the children clearer to themselves as well as those around them.

### Becoming Clearer to Oneself

Both affect awareness and tolerance increased significantly in the children with internalizing difficulties after therapy. This implied that they became better able to attend to and recognize affective states. In addition, their tolerance of affective experiences and their ability to cope with and regulate affect increased. These increases have important implications for development. When integrated, affect can function as a vitalizing and regulating force that firmly connects us with our own body, infusing events with personal meaning, significance, and urgency (Campos et al., 2004; Jacobsen and Svendsen, 2010). If, however, affect is poorly integrated, important information can be missed or misattributed as physical symptoms or non-specific unease or arousal. The experiential dimensions of affect integration, awareness, and tolerance are essential for affect’s adaptive and regulating potential. Increased awareness and tolerance can allow affective states to be not just noticed but also consciously and volitionally attended to, stayed with, and acted upon. This gives access to more meaningful information about the self and the outer world while also allowing affects to be dealt with in the conscious and interpersonal realm. Increases in affect awareness and tolerance make affective states more available to be both processed, regulated, and function as sources of information, regulation, and navigation. Thus, the children and significant events around them become more apparent and meaningful through more integrated affective experiences.

In light of the importance of affect in development and mental health (Sterling, 2012; Murphy et al., 2017), increases in affect awareness and tolerance represent a possible increase in psychological resilience in the face of stressors as well. In support of this, higher affective awareness can protect children from depressive reactions following interpersonal stress (Flynn and Rudolph, 2010). The increased affect awareness and tolerance in the children with internalizing difficulties could therefore continue to promote development, growth, and resilience beyond therapy.

### Becoming Clearer to Others

The ability to elicit adequate responses and have needs recognized and met by another is central to a child’s sense of self, agency, efficacy (Stern, 2000), and healthy child-caregiver transactions (Sameroff, 2009). In essence, our affective expressions tell others what we need, how badly we need it, and at what distance we want it. In this way, affect expression is essential for regulating interpersonal needs, intimacy, and boundaries. Particularly emotional (non-verbal) expressivity is vital for children and their caregivers, demonstrated by the fact that children come into the world ready to engage in complex, non-verbal emotional interactions right from the start (Tronick, 1989; Stern, 2000). The expressive dimensions of affect integration (emotional and conceptual expressivity) capture the ability to communicate affect, non-verbally, and with words. This ability is crucial for precise and efficient communication, bridging the divide between subjective inner realities and forming the basis of intersubjectivity.

The results from our study showed that the children with internalizing difficulties improved their ability to express and communicate affect significantly after TIC. This improvement included an increased capacity to acknowledge, recognize, decode, and express different non-verbal or verbal expressions of affect as assessed by the ACI. The results are supported by an earlier study of TIC where parents experienced their children as more emotionally expressive and easier to understand after therapy (Haugvik and Johns, 2008). The improvement in affect expressivity in the current study implies that the children became clearer to others, increasing their chances of receiving adequate attunement and developmental support after the conclusion of therapy. Parallel work with caregivers is essential in this respect. A central aspect of the parental work in TIC is the clarification and translation of the child’s affective signals, helping caregivers to see their children in a new or more nuanced light, strengthening their ability to reflect around and support their child (Haugvik, 2012).

The following anonymized example illustrates different ways of assessing and working with both children and caregivers in TIC.

### A Little Foal Wanting to Explore the World

One of the children in the internalizing group, *Emma* (aged 9), was referred with frequent, sudden bouts of crying and many psychosomatic symptoms. She was seen as more vulnerable, clingy, and less self-assertive than her peers. She often got into conflict with a sibling, and her parents had a hard time understanding and relating to Emma at the time of referral.

#### Pre-therapy Assessment of Emma

The pre-therapy assessment revealed that Emma’s mother had a history of mental health problems and severe depression that had been present since before Emma’s birth. Intersubjectivity with caregivers that suffer from psychopathology can lead to difficulties with affect integration and the development of symptoms, as the caregiver may not be sufficiently sensitive to the child’s feelings or emotional and regulatory needs. For instance, systematic failures to attune to certain sensations, impulses, or affects can lead them to be framed as outside of the realm of that which can be shared, tolerated, or regulated. Failures of attunement can also lead children to send affective signals aimed at what they believe the caregiver needs or expects rather than what the child experiences, leading to a “false” representation of self. At the same time, over-identification, or over-involvement from a caregiver in response to specific affects can overshadow or overpower a child’s genuine affective experiences and lead some affects to be dismissed or exaggerated (Tronick and Beeghly, 2011; Hansen, 2012). In line with this, Emma’s mother described frequent periods where she could not engage in attuned regulatory interactions with Emma from infancy, unable to even get out of bed. Expressions of negative affect in Emma often caused more negative affect in the mother. When the mother felt overwhelmed by negative affect, she would frequently cling to her daughter as a source of safety and support. The mother also struggled to separate from Emma or let Emma do activities alone or with others, often getting anxious or angry when Emma expressed an interest in doing things without her.

Based on this information, it was suspected that Emma had had little regulatory support in the face of negative affect and that she might have been frequently overwhelmed by both her own and her mother’s negative affect growing up. The developmental history also indicated a selective attunement away from signals of autonomy or exploration in Emma, instead reinforcing her signals of anxiety or need for help. It was observed that when Emma was with her parents, she seemed to shrink into the role of a younger and more helpless child than when she was alone with the therapist, indicated by a thin, squeaky voice and diminutive posture.

Pre-therapy assessment with the ACI indicated difficulties with most aspects of negative affect, along all dimensions (i.e., awareness, tolerance, and emotional and conceptual expressivity). The ACI indicated that negative affect was very difficult to both experience and adaptively express for Emma. Emma also scored low on the experience and expression of interest/excitement, enjoyment/joy, and tenderness/care, indicating difficulties staying with, regulating, making sense of, and expressing positive experiences and affects, as well as interpersonal closeness. These findings were understood in light of Emma’s developmental history and early interactions, with instances of possibly overwhelming negative affect, inadequate co-regulation, and little room for independent and joyful exploration. Based on the pre-therapy assessment and observations and interactions with Emma and her parents the following therapeutic focus was presented to Emma and her parents and agreed upon: “*Here we will work together to explore your hidden treasures.*”

#### Emma’s Therapy

It is an explicit aim in TIC to provide a safe space for the child and therapist to jointly attend to, experience, and express affective states in new ways, thereby allowing affect to become more meaningful and adaptive through new, corrective experiences. Based on her developmental history, it was considered crucial to give Emma experiences of having influence on her environment. Therefore, the therapist closely followed and attuned to Emma’s impulses, intentions, and affects, letting Emma experience that she could be understood and that it was both possible and safe to signal for and elicit behavior in another based on how *she* felt and what *she* needed. Implicit and non-verbal aspects of dialogue constitute essential building blocks of our internal representations of relational experiences from infancy and beyond (Stern, 2000) and can often reach where words cannot (Schore, 2019). Through non-verbal dialogue, new relational experiences can be integrated “bottom-up,” procedurally and implicitly, rather than through “top-down” cognitive-verbal processes. Because of this, body language, gaze, dwelling pauses, shared attention, and turn-taking were considered crucial aspects of the therapeutic dialogue with Emma. The therapist needed to attune closely to Emma, following her signals, impulses, and needs while monitoring and regulating important and salient themes in therapy. For the therapist, this entailed immersion in the intersubjective exchanges while simultaneously keeping a subjective eye on the process.

While sensitive attunement is essential in TIC, the therapist’s recognition of instances of less-than-optimal attunement and relational rupture are just as important, allowing the child to experience and participate in interactive reparations and the formation of new interactional models that include repair (Tronick et al., 1998; Tronick and Beeghly, 2011). The ability to adaptively face and navigate challenges at any stage in life may begin with the early dyadic ability to repair momentary ruptures in contact and attunement (Beeghly and Tronick, 2011). At the start of therapy, the expression of negative affect, disagreement, or self-assertiveness was difficult for Emma, and she took little initiative in therapy. However, at the end of treatment, Emma expressed disagreement or annoyance clearly and forcefully when the therapist misunderstood or misremembered something. Her expressions of disagreement or annoyance would prompt the therapist to repair the relationship, giving Emma an experience where her negative affect was seen and met without becoming overwhelming or relationally disruptive. Importantly, these episodes brought negative affects into the fold of potentially safe affects for interpersonal regulation. They also showed Emma that the relationship could survive her genuine expressivity, providing positive corrective experiences.

The importance of attunement, rupture, and repair in TIC is further confirmed by a micro-analysis of videos from a small subset of the therapies in the current study (Johns, 2018). These analyses showed that the therapists’ synchronization to the child’s signals contributed to increased clarity in the children’s affective communication. Noticing and adjusting to mismatches and errors in non-verbal emotional communication, such as tempo and intensity, as well as verbally, was particularly important. These findings highlight the importance of intersubjective and relational repairs in TIC. Importantly, the ability to repair relational rift is linked to better affect-regulation later in life (Kemp et al., 2016). In this way, experiences of rupture and consequent repair in therapy may provide further resilience and developmental growth beyond therapy as well.

Another focal part of therapy was increasing Emma’s tolerance for negative affect. Therefore, the therapist was very attentive to and reinforced Emma’s signals of negative affect in increasing doses deemed tolerable. It was important to allow Emma control of how much and when she wanted to access and share negative affect while also carefully expanding her range and tolerance through scaffolding and titration. Play is a forceful technique for scaffolding a child’s abilities and often allows for exploration of challenging themes less approachable through more direct routes. In line with this, play was a meaningful way for Emma to express and dwell on emotions she could not stay with in more direct conversation. The sharing of negative and positive affects like sadness, fear, joy, and curiosity during play made a more comprehensive range of affects shareable, at a safe distance and in “pretend-mode.” In the play, a salient theme was a baby foal trying to escape a nervous, overly fussy mother-horse, mirroring the dynamics between daughter and mother. The foal escaped the situation by burying the mother in the sandbox, leaving Emma seemingly relieved and happy afterward. In addition, air hockey and games of catch were used to help Emma regulate between more emotionally intensive parts of therapy and establish rhythm, contact, and reciprocity in the exchanges.

#### Work With Caregivers

Parallel work with caregivers is a crucial part of TIC. As the pre-therapy assessment indicated problematic transactions with selective attunement and little room for Emma to explore or become independent, the caregiver-therapist explored alternate and developmentally supportive ways for Emma’s parents to interact with their daughter. Emma’s difficulties were reframed in developmental themes, including attachment and need for protection and care vs. the need for exploration and autonomy. The father was encouraged to become more engaged and present, making the mother less likely to get overwhelmed by negative affect. As Emma was perceived as hard to read and understand at the start of therapy, it was considered vital to increase the parent’s ability to notice, attune to and meet Emma’s affective expressions appropriately. Through the parallel sessions, the parents developed their ability to notice, understand and contain Emma’s affective signals through reflective conversations with the caregiver therapist. Strengthening the mother’s ability to only fuss over Emma when Emma wanted closeness and to allow Emma more autonomy to explore by herself was considered equally important.

#### Post-therapy Assessment of Emma

At the end of therapy, Emma’s symptoms had subsided considerably, normalizing her CBCL-scores. She cried much less, and she seemed happier and more visible to herself and those around her. She was also described as better able to notice, tolerate, and use affect as a source of information about what she wanted and needed. She was seen as more precise and transparent in her expression of different affects and desires. For example, she could now use anger to set explicit boundaries with others, becoming more assertive and independent than before therapy. In support of the clinical observations, the post- ACI showed increased affect integration for all nine affective categories across the experiential (awareness and tolerance) and expressive (emotional and conceptual) dimensions. Affects anger/rage, interest/excitement, enjoyment/joy, and tenderness/care improved the most, echoing her increased ability to set boundaries for herself, her increased autonomy and independence, and her seemingly more genuine relationship with her family. Likewise, her parents seemed better able to recognize, attune to, and meet Emma’s feelings, and the mother proudly supported her daughters’ activities outside of the home. As Emma became more apparent to herself and others, and her parents became better able to see and meet her, their transactions changed, appearing more age-appropriate and developmentally supportive. It was concluded that Emma and her family were on a healthier developmental track, with no need for further therapy.

### Time-Limited Intersubjective Child-Psychotherapy as a Right-Brain Psychotherapy Approach Targeting Implicit Processes and Common Factors

Schore (2019) differentiates between predominantly left-brain oriented psychotherapy approaches targeting language-based or cognitive processes and predominantly right-brain oriented approaches targeting emotional and implicit processes. Schore’s concept of right-brain psychotherapy builds on work by Trevarthen on intersubjective exchanges and developmental laterality (Trevarthen, 1993, 1996; Trevarthen and Aitken, 2001), attachment theory (Schore and Schore, 2008), and psychodynamic theory (Schore, 2019). So far, much of psychotherapy research has focused on pre-dominantly left-brain oriented therapies trying to affect change through language and cognitive processes. such as cognitive therapy. While more left-brain oriented therapy is sufficient for some patients, when problems are centered on emotional dysregulation rooted in early development a right-brain oriented psychotherapeutic approach is necessary to affect real change. Right-brain psychotherapy operates through implicit, non-verbal intersubjective exchanges between the therapist and patient, where “rapid communications between the right-lateralized “emotional brain” (“right mind”) of each member of the therapeutic alliance allow for moment-to-moment, right brain-to-right brain “self-state sharing,” a co-created, organized, dynamically changing dialogue of mutual influence” (Schore, 2019). Further, in this intersubjective state-sharing each participant “match[es] the dynamic contours” (Schore, 2019) of the arising emotional-motivational states of the other, while synchronizing and adapting their own attention and arousal in response to the other’s increasing and decreasing, accelerating and decelerating, implicit, non-verbal signals. This corresponds to a matching and synchronization between patient and therapist in what Stern labeled vitality affects (Stern, 2000; Sørensen, 2006), constituting the core of our sense of embodied aliveness. The transactions between child and therapist are also in line with Tronick’s expanded states of consciousness model (Tronick et al., 1998), and a view of cognition (Clark, 2008), and self (Bolis and Schilbach, 2020) as both embodied and contextually and relationally embedded. Importantly, the intersubjective sharing provides the patient with new corrective experiences of feeling seen, met, and functioning in a state of higher self-organization and regulation possible than when alone. These new corrective experiences may include experiences of regularity and safety, implicit affective sharing, and repair of relational rupture, all processes important for development (Sørensen, 2006; Schore and Schore, 2008). Based on TIC’s explicit focus on implicit and affective intersubjective processes within a regulating and regular framework, we argue that TIC is a prime example of right-brain psychotherapy consistent with Trevarthen’s (Trevarthen, 1993; Trevarthen and Aitken, 2001) and Schore’s work (Schore, 2019, 2021).

Furthermore, much in the same way left-brain oriented approaches have received more attention than right-brain oriented approaches, so-called non-specific common factors in psychotherapy have received less attention compared to specific treatments or techniques (Laska et al., 2014). Common factors believed to operate in all effective therapeutic approaches include therapist empathy, therapeutic alliance, and goal consensus (Laska et al., 2014). An investigation of overall effect sizes from psychotherapy research indicates that these common factors are likely more important than specific techniques and should be considered part of evidence-based treatment in their own right (Laska et al., 2014). We argue that TIC is not only a right-brain psychotherapy providing salient and enveloping experiences of intersubjective, right-lateralized co-regulation, but also a therapeutic approach well suited to potentiate common factors, specifically experiences of therapist empathy, alliance, and goal consensus. Firstly, the explicit therapeutic stance of pervasive and sensitive child-centering in TIC is likely related to perceived empathy through, for instance, inter-personal synchronization (Wiltshire et al., 2020). Secondly, the structure of TIC is specifically designed to increase alliance and goal consensus (Johns and Svendsen, 2016). A main purpose, and effect, of conducting therapy within a time-limited frame is the idea that the clear and bounded time-frame will help expediate the forming of a working alliance, as opposed to an open-ended, less clear process (Mann, 1991). Furthermore, an intersubjective dialogue may facilitate the negotiation of different narratives around challenges, therapy, and stigma, as shown in an intersubjective approach to the treatment of schizophrenia (Hasson-Ohayon et al., 2017). The negotiation and navigation of different perspectives and narratives is a salient issue in child psychotherapy, considering many children come to therapy because of the concern of others, not necessarily of their own volition. In addition, in TIC the therapeutic focus and the importance of following the child’s attention in the here-and-now is aimed at providing a strong and rapidly forming alliance, as well as ensuring that goals and tasks are mutually accepted and understood. This includes ensuring that the child and parents explicitly agree working on the focus, expanding the therapeutic alliance to include both the child and parents. Interestingly, the fact that the focus is formulated as a metaphor may not only contribute to the child’s motivation and understanding of therapy, thereby strengthening the alliance and goal-consensus, but may also function as a bridge between left-lateralized semantic language processes and right-lateralized emotional processes due to the relative right-lateralization of metaphors (Schore, 2019).

### Limitations and Constraints on Generalizability

The study had a small, culturally homogenous sample size and did not use a staggered-entry design with an internalizing control group, instead relying on healthy controls. These limitations mean that the ability to make conclusions about causality or generalizability is limited, and the results must be considered preliminary, and in need of replication in a study with a control group with internalizing psychopathology. A further limitation is that we did not assess verbal intelligence or working memory, factors that may impact the capacity for affect integration (Lindquist and Barrett, 2008; Taarvig et al., 2015). Finally, it is a limitation that we did not observe the changes in affect integration over time or with multiple instruments as this would have improved the rigor (Cole et al., 2004).

## Conclusion

One of the defining features of time-limited intersubjective child psychotherapy is the focus on supporting development and on right-lateralized, implicit, intersubjective sharing of emotional-motivational states. Affect integration is important for a child’s sense of self and others and for communication of needs and intentions. Difficulties with affect integration may lead to experiences of disruptive arousal that seemingly lack motivation and meaning, making the child appear unclear to others and itself. Difficulties with affect integration may also contribute to internalizing symptoms and dysregulated transactions with others. After 10 h of TIC, children with internalizing difficulties showed decreased internalizing symptoms and increased affect integration overall and in terms of awareness, tolerance, and emotional and conceptual expressivity. Increased affect awareness meant the children were better able to recognize and attend to mental and physical signs of affect. The increases in affect tolerance meant they were better able to tolerate and make sense of affect, allowing for more adaptive regulation. The increased ability to communicate affect non-verbally and verbally meant the children’s chances of eliciting appropriate developmental support from others also improved. We argue that TIC represents a prime example of a right-brain psychotherapeutic approach that profits from a strong focus on enhancing common factors such as alliance and goal consensus. The results from this study show that TIC may reduce symptoms of internalizing disorder, and perhaps more importantly, strengthen affect integration, which in turn can help guide children with internalizing difficulties and their caregivers onto healthier developmental trajectories facilitating further growth.

## Data Availability Statement

The raw data supporting the conclusions of this article will be made available by the authors, without undue reservation.

## Ethics Statement

The studies involving human participants were reviewed and approved by Regional Committees for Medical and Health Research Ethics, division North. Written informed consent to participate in this study was provided by the participants’ legal guardian/next of kin. Written informed consent was obtained from the minor(s)’ legal guardian/next of kin for the publication of any potentially identifiable images or data included in this article.

## Author Contributions

TA and CF performed the analyses. CF wrote the manuscript and prepared the submission. TA, UJ, and KJ read the manuscript and gave critical feedback. KJ was the principal investigator. UJ contributed as a therapist. All authors contributed to the initial planning and execution of the study and approved the submitted version.

## Conflict of Interest

The authors declare that the research was conducted in the absence of any commercial or financial relationships that could be construed as a potential conflict of interest.

## Publisher’s Note

All claims expressed in this article are solely those of the authors and do not necessarily represent those of their affiliated organizations, or those of the publisher, the editors and the reviewers. Any product that may be evaluated in this article, or claim that may be made by its manufacturer, is not guaranteed or endorsed by the publisher.
